# Depression in public officials during the COVID-19 pandemic in Paraguay: a web-based study

**DOI:** 10.1186/s12889-021-11860-z

**Published:** 2021-10-11

**Authors:** Ji Eon Kim, Ji Ho Lee, Yanghee Kang, Sun Ha Lee, Hyein Shin, Nadia Rönnebeck, Renato Rönnebeck, Eun Woo Nam

**Affiliations:** 1 Department of Health Administration, Graduate School, Yonsei University, Wonju, Republic of Korea; 2 Yonsei Global Health Center, Wonju, Republic of Korea; 3 Department of Health Administration, College of Health Science, Yonsei University, Wonju, Republic of Korea

**Keywords:** COVID-19 pandemic, Depression, Mental health, Self-quarantine, Paraguay

## Abstract

**Background:**

According to the World Health Organization, the coronavirus disease 2019 (COVID-19) pandemic has created situations that have a negative effect on people and threaten their mental health. Paraguay announced the Estado de Emergencia Sanitaria (Presidential Decree No. 3456) on March 16, 2020, which was followed by the imposition of a 24-h restriction on movement order on March 21. Self-quarantine at home may have been the most effective method of preventing the spread of infectious diseases; however, with the global pandemic becoming more prolonged and the consequent lengthening of the 24-h self-quarantine period, it is highly probable that both physical and psychological problems will arise.

**Methods:**

In this study, a web-based cross-sectional method was used to analyze the factors influencing COVID-19-induced depressive feelings in Paraguayan public officials.

**Results:**

Public officials reported a high level of depressive symptoms with a high level of apprehension in early stage of COVID-19. In addition, this study identified that when the self-quarantine period increased, levels of depressive feelings also increased. Since self-quarantine is characterized by the requirement that individuals endure an undetermined period within a confined area, it may have caused stress and anxiety, as well as the consequent experience of depressive feelings.

**Conclusions:**

Paraguayan government should develop a program for the delivery of mental health care and services to public officials in COVID-19 Pandemic period. Moreover, a program is required for people facing deteriorating mental health due to social isolation and loneliness caused by social distancing during the prolonged period of self-quarantine. Finally, mental health care programs should be organized in a community-focused way by utilizing online systems to enhance the effectiveness of mental health recovery.

## Background

Coronavirus disease 2019 (COVID-19) first emerged in Wuhan City, Hubei Province, China, at the end of 2019, and rapidly spread worldwide, continuing even in 2020. The World Health Organization (WHO) declared the COVID-19 outbreak as a phase VI pandemic—the highest alert level—on March 11, 2020 [1]. The disease spread throughout South America, with the first confirmed case on February 26, 2020, involving a patient returning to Brazil from northern Italy [2]. In Paraguay, the first confirmed case involved a patient who returned from Ecuador on March 7, 2020. The cumulative number of confirmed cases was 208 as of April 20, 2020, with eight deaths, indicating a 3.85% fatality rate for COVID-19 in Paraguay [3]. The number of confirmed COVID-19 cases is smaller in Paraguay than in its neighboring countries; however, being a land-locked nation with substantial influence from Brazil and Argentina, its number of confirmed patients has steadily increased [4].

According to the WHO, the COVID-19 pandemic has created situations that negatively affect people and threaten their mental health [5]. The Centers for Disease Control and Prevention (CDC) stated that the circumstances created by COVID-19 were likely to induce high levels of fear, anxiety, and stress, which may develop into depression and deteriorate people’s psychological health [6]. A nationwide survey performed by the American Psychiatric Association in the United States reported that over one-third of American citizens were psychologically affected by COVID-19 [7]. The Mayo Clinic stated that during the COVID-19 pandemic, people who experience changes in their daily activities, social isolation due to quarantine, stress, loneliness, and anxiety could develop mental health disorders such as depression [8].

The province of Quebec in Canada established a program to manage the stress, anxiety, and depression that may occur during self-quarantine due to COVID-19 [9]. According to the Director of the University of Washington’s Center for the Science of Social Connection, Mr. Jonathan Kanter, social isolation, turmoil, and extreme changes in daily activities caused by COVID-19 are highly likely to cause clinical depression. Further, medical staff and people engaged in work that directly deals with COVID-19 face even more serious threats to their psychological health [10]. The British Broadcasting Corporation, UK and the University of Washington’s Center for the Science of Social Connection, US have predicted that the compulsory long-term quarantine and social distancing that governments use as basic strategies against COVID-19 have a high probability of inducing social isolation and loneliness, which could develop into depression, with women being more vulnerable to depressive symptoms [11, 12].

**Fig. 1:**
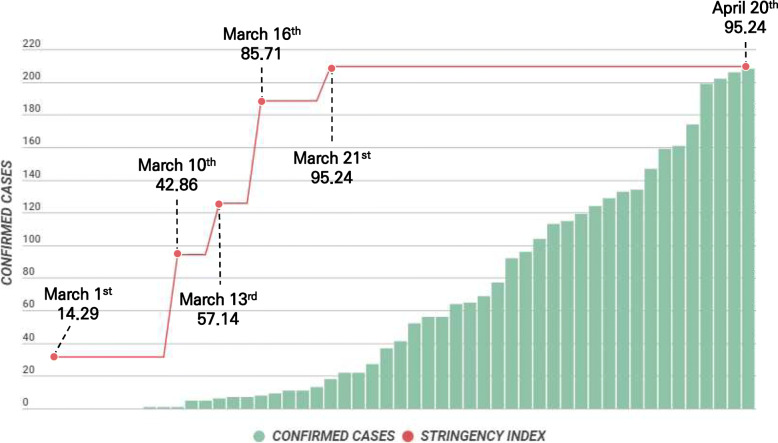
Current status of the COVID-19 Government Stringency Index and confirmed cases in Paraguay^1)^. ^1)^ DATA 2020-04-20

Paraguay announced the *Estado de Emergencia Sanitaria* (Presidential Decree No. 3456) on March 16, 2020, which was followed by the imposition of a 24-h restriction of movement order on March 21 [13]. The Oxford COVID-19 Government Response Stringency Index (Fig. 1) shows that the government in Paraguay has implemented strong COVID-19 response policies since March 21, with a mean score of 95.24 out of 100 [14]. Self-quarantine at home is perhaps the most effective method of preventing the spread of infectious diseases [15]. However, with the global COVID-19 pandemic getting prolonged and the consequent lengthening of the 24-h self-quarantine period, it is highly probable that both physical and psychological problems will arise. Based on an online application, the government of Paraguay distributed self-quarantine guidelines for people who found self-quarantine considerably difficult and also made efforts to minimize the negative impact of self-quarantine. Therefore, the present study aimed to provide scientific evidence to support the Paraguayan government’s policies while also offering fundamental data.

This study was conducted using the COVID-19 online survey tool developed by the Yonsei Global Health Center (YGHC). Factors influencing depressive feelings in Paraguayan public officials caused by the COVID-19 pandemic were investigated using a survey questionnaire.

The association of COVID-19 awareness and the factors influencing the COVID-19 pandemic with the depressive feelings in Paraguayan public officials were identified.

## Methods

### Study design

This study used a web-based cross-sectional method to analyze the factors influencing COVID-19-induced depressive feelings in Paraguayan officials. Its research area was Asuncion and the Central Limpio in Paraguay.

### Materials

This study used the YGHC COVD-19 Online Survey Tool developed by the Yonsei Global Health Center. The survey questions were selected through expert meetings comprising public health experts at the Yonsei Global Health Center (YGHC), and content validity was confirmed through pre-tests in 10 individuals. The survey questionnaire covered sociodemographic characteristics, symptoms and physical health status, patients’ contact history, precautionary measures against COVID-19 in the past 14 days, information related to COVID-19, and the Patient Health Questionnaire (PHQ-9). The PHQ-9 was developed to diagnose major depressive disorder and consists of nine questions. The PHQ-9 is known to be superior to previous diagnostic tools for depression in terms of reliability and time for completion [16]. The internal reliability of PHQ-9 is good, with a Cronbach’s α of 0.89. We had original English questionnaire, and translated into 5 different languages: Korean, Chinese, Japanese, French and Spanish by foreign researcher in our center. The questions were reviewed by local professors and experts in each country to produce questionnaires that are suitable for each country.

### Data collection

The subjects in this study were officials aged ≥20 years, who were central government employees at Asuncion and at the Central Limpio in Paraguay. For information extraction, a snowball sampling technique, in which the existing study subjects recruit future subjects from among their acquaintances, was used. An online questionnaire using Google Forms was distributed to the heads of departments in Paraguay’s central government. The heads who received the questionnaire conducted a survey by passing an “online questionnaire link” to other Paraguayan officials through SNS (WhatsApp). Then, the officials distributed the “link” to other acquaintances by SNS and used the method to broaden the target group. The researcher was able to check the number of people who completed the survey in real time. While there were no dropouts or missing values in the data, 27 respondents who did not satisfy the subject criteria were excluded, and a total of 171 respondents were selected as the study subjects. The survey period was from April 32,020 to April 17, 2020 (approximately 2 weeks).

### Variables

#### Dependent variables

In this study, each subject’s score was calculated using the PHQ-9 consisting of nine items corresponding to the diagnostic criteria of the Diagnostic and Statistical Manual of Mental Disorders, Fourth Edition, which is a screening tool for depression that indicates how frequently a participant has suffered from the given problem in the past 2 weeks [17]. Although the PHQ-9 has not yet been officially used in Paraguayan studies, it has been applied in reports and studies that investigated the factors influencing depression in Central and South American countries, including Brazil, Argentina, and Peru. Urtasun et al. (2019) reported that for Argentina, the optimal cut-off score for the PHQ-9 was ≥8 (sensitivity, 88.2%; specificity, 86.6%; positive predictive value, 90.91%) [18].

#### Independent variables

The independent variables in this study were demographic characteristics such as sex, age, and educational, marital, and subjective health status. COVID-19 awareness was analyzed based on the past 14 days’ usage of the following independent variables: number of times precautionary actions against COVID-19 had been practiced, apprehensions about severe acute respiratory syndrome coronavirus 2 (SARS-CoV-2) infection, and period of self-quarantine due to COVID-19. The respondents’ data were collected at the time of the survey. Age was treated as a continuous variable, and sex was categorized as *male* or *female*. Educational status was divided into *undergraduate* and *graduate or higher,* marital status into *married* and *unmarried,* and subjective health status as *not bad* and *good* for the subsequent analyses. For COVID-19 awareness, the independent variables were subdivided, as shown in Table 1. To calculate the number of precautionary actions against COVID-19 practiced during the past 14 days, those who had answered “practice most actions” were classified as “*compliant*.” With regard to apprehensions about COVID-19 during the past 14 days, the responses received comprised three categories: “*never worried,” “occasionally worried,” and “constantly worried*.” Lastly, for the period of self-quarantine due to COVID-19 during the past 14 days, the responses took the form of units of time.

**Table 1:** Summary of the independent variables (COVID-19 awareness)

Variables	Questionnaire
**COVID-19 precautionary actions practiced during the past 14 days**	1) Covering one’s mouth when coughing/sneezing
	2) Avoiding public transportation
	3) Washing hands using soap and water
	4) Washing hands after sneezing
	5) Wearing a face mask at all times
	6) Washing hands after touching a contaminated object
	7) Refraining from using an elevator
	8) Refraining from attending meetings of ≥10 individuals
**Apprehensions about COVID-19 infection during the past 14 days**	1) Never worried
	2) Occasionally worried
	3) Constantly worried
**Period of self-quarantine due to COVID-19 during the past 14 days**	Unit of time

### Statistical analysis

The factors influencing depressive feelings in public officials in Paraguay due to the COVID-19 pandemic were analyzed using SPSS 25.0, following the methods detailed below.

First, a correlation analysis was carried out to analyze the relationships among the general characteristics, COVID-19 awareness, and depressive feelings in the study subjects.

Second, a hierarchical multiple regression analysis was performed to analyze the influence of COVID-19 awareness on depressive feelings in the study subjects.

## Results

### General characteristics of the study subjects

The general characteristics of the study participants are presented in Table 2. Regarding sex distribution, the number of women (60.2%) was greater than that of men (39.8%). The subjects’ average age was approximately 34 years, with those in their 30s accounting for 52.6%, followed by 24.0% in their 20s, and 23.4% in their 40s or above. Regarding educational and marital status, 85.4% were undergraduates and 14.6% were graduates or higher; 71.3% were unmarried, and 28.7% were married. Regarding subjective health status, those who responded ‘*not bad’* constituted 52.6%, being higher than those who responded ‘*good’* (47.4%).

**Table 2:** General characteristics of the study subjects. *n* = 171(%)

Variables	Category	n(%)
**Sex**	Male	68(39.8)
	Female	103(60.2)
**Age** (34.36 ± 7.589)	20s	41(24.0)
	30s	90(52.6)
	40s or above	40(23.4)
**Educational status**	Undergraduate	146(85.4)
	Graduate or higher	25(14.6)
**Marital status**	Unmarried	122(71.3)
	Married	49(28.7)
**Subjective health status**	Not bad	90(52.6)
	Good	81(47.4)

### Correlations among the main variables

The analysis revealed that women were more likely to have apprehensions about COVID-19 infection than men (r = .169, *p* < .05). People with good subjective health were more likely to have shorter self-quarantine periods due to COVID-19 than those who responded badly (r = −.151, p < .05). According to the relationship between depression (PHQ-9) and variables, it was found that worry about COVID-19 infection is associated with a higher PHQ-9 score (r = .311, *p* < .01). Women were more likely to feel depressed than men (r = .242, *p* < .01), and it was found that unmarried people were more likely to feel depressed than married people (r = .196, *p*<. 05). Those who responded that they had good subjective health were less likely to feel depressed than those who did not (r = −.282, *p* < .01), and it was found that the older they were, the higher was the likelihood of their depression scores being lower (r = −. 219, p < .01). Finally, the longer the self-isolation period due to COVID-19, the higher the likelihood of feeling depressed (r = .049, p < .01) (Table 3).

**Table 3:** Correlations among the main variables. n = 171(%).

Variables	(1)	(2)	(3)	(4)	(5)	(6)	(7)	(8)	(9)
**Sex (1)**	1								
**Age (2)**	−.024	1							
**Educational status (3)**	−.099	−.173^*^	1						
**Marital status (4)**	.093	−.370^**^	.140	1					
**Subjective health status (5)**	−.091	−.024	−.171^*^	−.098	1				
**Number of times** **precautionary measures against COVID-19 were practiced (6)**	−.089	−.113	.097	−.046	−.037	1			
**Apprehensions about COVID-19 infection (7)**	.169^*^	.006	−.014	.032	−.145	−.089	1		
**Period of self-quarantine due** **to COVID-19 (8)**	.097	−.066	.117	.181^*^	−.151^*^	−.015	.057	1	
**Depressive feelings (PHQ-9) (9)**	.242^**^	−.219^**^	.084	.196^*^	−.282^**^	.060	.311^**^	.049^**^	1

*p < .05, **p < .01, ***p < .001

### Association between depressive feelings and the self-quarantine period due to COVID-19

The results of the factors influencing depressive feelings are presented in Table 4. A hierarchical multiple regression analysis was carried out to analyze the relationship between COVID-19 awareness and depressive feelings, and a four-step regression analysis was carried out to control the subjects’ general characteristics and determine the unique influence of COVID-19 awareness. First, the variance inflation factor (VIF) index was used to examine the multicollinearity among the independent variables, and the results showed that their VIF was 1.158–2.891, indicating the absence of multicollinearity. In addition, testing the independence of errors showed that the Durbin-Watson statistic was 1.818, indicating its lack of autocorrelation and suitability as a regression model.

**Table 4:** Association between the period of self-quarantine due to COVID-19 and depressive feelings. n = 171(%)

Depressive feelings (PHQ-9)								
Variables	Model 1		Model 2		Model 3		Model 4	
	*β*	*t(p)*	*β*	*t(p)*	*β*	*t(p)*	*β*	*t(p)*
**Sex**								
Male	(ref)		(ref)		(ref)		(ref)	
Female	.208	2.911^**^	.209	2.911^**^	.176	2.511^**^	.149	2.283^**^
**Age**	−.187	−2.441^**^	−.183	−2.368^**^	−.179	−2.368^**^	−.179	−2.592^**^
**Educational status**								
Graduate or higher	(ref)		(ref)		(ref)		(ref)	
Undergraduate	.018	.239	.014	.190	.009	.125	−.019	−.292
**Marital status**								
Married	(ref)		(ref)		(ref)		(ref)	
Unmarried	.080	1.038	.083	1.073	.080	1.072	.030	.428
**Subjective health status**								
Not bad	(ref)		(ref)		(ref)		(ref)	
Good	−.257	−3.554^**^	−.255	−3.517^**^	−.217	−3.079^**^	−.180	−2.726^**^
**Number of times precautionary measures against COVID-19 were practiced**			.040	.562	.070	1.010	.078	1.205
**Apprehensions about COVID-19 infection (in the past 14 days)**								
Never worried					(ref)		(ref)	
Occasionally Worried					.072	.621.	.084	.782
Constantly worried					.325	2.778^**^	.323	2.972^**^
**Period of self-quarantine due to COVID-19 (in the past 14 days)**							.335	5.135^***^
**F**	7.303^***^		6.113^***^		6.839^***^		9.961^***^	
**R**^**2**^	.181		.183		.252		.358	
**Adjusted R**^**2**^	.156		.153		.216		.322	
**VIF**	1.158–2.891							
**Durbin-Watson**	1.818							

*p < .05, **p < .01, ***p < .001

Model 1 was based on the first input of variables after controlling for general characteristics of the study subjects, and the results indicated 18.1% explanatory power with statistical significance. The number of times precautionary actions against COVID-19 had been practiced was added to model 1 as a new variable in model 2, and the explanatory power was 18.3%, without a significant change. In model 3, the apprehensions about SARS-CoV-2 infection variable during the past 14 days was additionally analyzed, leading to a statistically significant result, with the explanatory power increasing to 25.2%. The result of the analysis in model 4—the final model—where the period of self-quarantine due to COVID-19 during the past 14 days was added as a variable to complete the model, showed an explanatory power of 35.8%. Among the study subjects’ general characteristics, sex (β = .149, *p* < .01), age (β = −.179, p < .01), and subjective health status (β = −.180, p < .01) were found to have a significant influence. The results indicated a higher level of depressive feelings in women than in men, and a lower level of depressive feelings as the subjects’ age increased. Furthermore, compared to subjects who responded ‘*not bad’* regarding their subjective health status, those who responded *‘good’* were found to have fewer depressive feelings. Among the COVID-19 awareness variables, the number of times precautionary measures against COVID-19 had been practiced did not exhibit statistical significance, whereas for apprehension about SARS-CoV-2 infection during the past 14 days, those who had responded ‘*constantly worried’* (β = .323, *p* < .01) showed higher scores for depressive feelings than those who responded ‘*never worried’*. As the self-quarantine period due to COVID-19 during the past 14 days increased, the scores for depressive feelings also increased and were significant (β = .335, *p* < .001).

## Discussion

There are various studies on the COVID-19 pandemic discussing depression in office workers and risk factors. Based on this paper, public officials reported a high level of depressive symptoms with a high level of apprehension in early stage of COVID-19. In addition, being in self-quarantine was associated with higher health anxiety and fear.

According to previous research, if public officials had depressive feelings in COVID-19 pandemic, it had likely influenced such as anxiety about the future, loss of joy and interest in frequently performed activities, financial difficulties, and exposure to false information [19]. With the COVID-19 pandemic getting prolonged, public officials in Paraguay may have experienced particularly heightened levels of anxiety, which may have caused a high level of depressive feelings. The WHO has also stated that as people’s daily activities undergo sudden changes due to COVID-19, their stress levels increase, and it is highly probable that this stress could develop into high levels of depressive feelings [20]. In Paraguay, a non-government organization implemented a support program using Facebook for people vulnerable to COVID-19, but no such programs exist to provide psychological support to public officials and medical staff managing COVID-19. Thus, to effectively respond to the prolonged COVID-19 pandemic, along with its stringent COVID-19 response policies, the Paraguayan government should develop a program for the delivery of mental health care and services to public officials.

The results of model 4 (see Table 4) showed that when the period of self-quarantine due to COVID-19 increased, experiencing a higher level of depressive feelings also increased. Since self-quarantine due to COVID-19 is characterized by the need for individuals to endure an undetermined period within a confined area, it may cause stress and anxiety and the consequent experience of depressive feelings. This implies the need for a program that can minimize mental health deterioration due to social isolation and loneliness caused by social distancing during the self-quarantine period. Therefore, many countries have implemented policies to connect people in society who have been affected by the COVID-19 pandemic via mental health care support groups. In addition, the WHO recommended starting physical activity during self-quarantine and emphasized activities such as “taking short active breaks during the day,” “following an online exercise class,” “walking,” “standing up,” and “relaxing” [21]. Mental and physical health care should be provided side by side to combat the high levels of depressive feelings during self-quarantine. Instead of waiting at home until the end of the COVID-19 pandemic, if individuals take light outdoor walks and embrace home-training to maximize their physical activities, together with strict observation of personal hygiene practices such as handwashing, it will surely make enduring the self-quarantine less painful. For individuals, a prolonged period of social distancing results in a fall in social capital, and it is crucial to restore such social capital. The best way to restore the social network and ensure its steady maintenance is the use of an online system. The online systems recommended by the CDC include telephone calls, e-mails, mail letters or cards, text messages, video chats, and social media [6]. The psychological effects of COVID-19 quarantine programs are likely to be maximized if they are run in a community-focused way, through which the blind spots of COVID-19 mental health care can be addressed. Among the Central and South American countries, Paraguay has ensured the reliable management of COVID-19, and its government has imposed stringent policies to meet its primary goal of preventing the spread of the pandemic. In this unprecedented global pandemic, Paraguay might become an even more successful model country for the management of COVID-19 if its future policies reflect the findings regarding the side effects of self-quarantine and social distancing that affect mental health.

### Limitations

This study had a few limitations. First, due to the COVID-19 pandemic, this study was conducted as a non-face-to-face web-based survey. Web-based surveys are available only to populations who can access the internet and read, so there may be biases in the sampling method. Second, as the study was conducted early in the COVID-19 pandemic, it is likely that depressive symptoms were much more severe due to several external factors for COVID-19.

## Conclusions

The Paraguayan government should develop a program for the delivery of mental health care and services to public officials in COVID-19 Pandemic period. Moreover, a program is required for people facing deteriorating mental health due to social isolation and loneliness caused by social distancing during the prolonged period of self-quarantine. Finally, mental health care programs should be organized in a community-focused way by utilizing online systems to enhance the effectiveness of mental health recovery. If the Paraguayan government thoroughly analyzes the side effects of the COVID-19 self-quarantine and social distancing and includes these findings in its future policies, Paraguay can become a successful model country for the management of the COVID-19 pandemic.

## Data Availability

The datasets used and/or analyzed during the current study are available from the corresponding author and can be released upon reasonable request.

## Abbreviations

COVID-19: Coronavirus disease 2019
WHO: World Health Organization
CDC: Centers for Disease Control and Prevention
US: United States
UK: United Kingdom
YGHC: Yonsei Global Health Center
PHQ-9: Patient Health Questionnaire
VIF: Variance Inflation Factor
